# No Environment, No Education: Honoring the Clinical Learning Environment as the Engine of Competency-Based Education – A Call to Action

**DOI:** 10.5334/pme.2548

**Published:** 2026-09-09

**Authors:** Eusang Ahn, Kelly J. Caverzagie, Arvin Damodaran, Soeren Huwendiek, Kimberly Lomis, Adrian P. Marty, David A. Turner, Teri Turner, Adelle Atkinson, As members of the International Competency-Based Health Professions Educators Collaborative

**Affiliations:** 1 Department of Emergency Medicine, University of Ottawa, Ottawa, Canada; 2 Oregon Health & Science University, Portland, USA; 3 School of Clinical Medicine, University of New South Wales, Sydney, Australia; 4 Department for Assessment & Evaluation, Institute for Medical Education, University of Bern, Bern, Switzerland; 5 Medical Education Innovations, American Medical Association, Chicago, USA; 6 Department of Anesthesiology, Intensive Care Medicine and Pain Therapy, University Hospital Balgrist, Zurich, Switzerland; 7 Innovation and Competency-Based Education, American Board of Pediatrics, Chicago, USA; 8 Baylor College of Medicine, Houston, USA; 9 Department of Paediatrics, University of Toronto, Toronto, Canada

## Abstract

Competency-Based Health Professions Education (CBHPE) has transformed health professions training by emphasizing demonstrated competence over time-based progression. Yet the clinical learning environment (CLE), the social, cultural, and organizational ecosystem in which learning occurs, has not evolved in parallel. Persistent hierarchies, workload pressures, and inadequate faculty development limit CBHPE’s full potential. Thus, the authors sought to identify priorities for CLE reform. A structured consensus process was conducted at the International Competency-Based Education Collaborators Summit in February 2025, where interdisciplinary health professions educators identified CLE reform priorities. Findings were refined through virtual meetings over three months. Five interdependent priorities were identified as essential for transforming the CLE:

- **Psychological safety:** enabling vulnerability, feedback, coaching, and growth through faculty and learner development
- **Teaching environment:** prioritizing faculty well-being, recognition, and sustainable teaching capacity
- **Desirable difficulty and productive struggle:** balancing challenge and safety to promote adaptive expertise and resilience
- **Interdependence and collectivism:** reframing education from individual performance to team-based competence and shared accountability
- **Partner alignment:** integrating educational and healthcare system goals to advance patient care and workforce well-being

Together, these domains outline the CLE as both a developmental and operational engine for CBHPE. Realizing the promise of CBHPE depends on transforming the CLE into a psychologically safe, appropriately challenging, interdependent, and system-aligned ecosystem. Reform demands cultural change that values coaching, feedback, vulnerability, and collective responsibility as professional imperatives. By aligning structures and empowering adaptive educators, the CLE can evolve from a passive setting to an active driver of successful competency-based transformation.

## Introduction

The core principles of competency-based health professions education (CBHPE), including outcome competencies, sequenced progression, tailored learning experiences, competency-focused instruction, and programmatic assessment, have led to sweeping changes in curriculum design and assessment [1,2,3,4]. However, the clinical learning environment (CLE), the setting in which the development of competence largely occurs, still requires significant overhaul to be optimized for CBHPE, specifically with regards to competency development, whether in the post-graduate residency environment, or within undergraduate medical schools.

Defined as “the social interactions, organizational cultures and structures, and physical and virtual spaces that surround and shape ‘participants’ experiences, perceptions, and learning,” the CLE functions as the delivery engine through which the core components of CBHPE are operationalized [3,5,6]. This relationship is particularly important because many of the defining features of CBHPE, including tailored learning experiences, longitudinal developmental assessment, meaningful coaching relationships, and continuous feedback, depend on workplace environments that support vulnerability, observation, reflection, and growth. When these conditions are absent, CBHPE risks becoming a compliance exercise focused on documentation and assessment processes rather than learner development [7].

The CLE in which one trains has a lasting imprinting effect on subsequent physician practice outcomes [8]. The U.S. Accreditation Council for Graduate Medical Education (ACGME) acknowledged this important influence through its Clinical Learning Environment Review (CLER) program [9]. From 2012–2025, the ACGME conducted site visits to evaluate key dimensions of the health systems in which training occurs, such as patient safety and healthcare quality. While there are differences globally, most CLEs, reflecting external and internal pressures, still uphold traditional norms of hierarchical structures, disciplinary silos, and problematic social and cultural environments that are not conducive to optimal learning [10,11].

The reasons for this gap are numerous and include broad external system issues, cultural inertia, ineffective faculty and learner development to lead change, lack of evidence-informed assessment practices, high service demands of the teams, poorly designed clinical models of care, and concerns about psychological safety [6,10,12,13,14,15]. This is not the first time the challenge of the CLE has been addressed globally [11]. Over the past two decades, efforts to improve the CLE have evolved from measurement and benchmarking initiatives [16] toward broader culture change efforts, including competency-based educational reform [17] and health-promoting learning environment frameworks [13], reflecting growing international recognition that learning environments are fundamental determinants of both educational and patient outcomes [14]. Through the CLER program, training programs and health system administrators received formative feedback to strengthen learning environments across health professions and the educational continuum [9]. The National Collaborative for Improving the Clinical Learning Environment framework in the United States focuses on safety, teaming, professionalism, and well-being [18,19] and the MaineHealth Interprofessional Partnership to Advance Care and Education (iPACE) model [20] seeks to improve team collaboration and learner satisfaction. Community of Practice forums and Deliberately Developmental Organization theory support institutional culture change and professional identity development as key to improving the CLE [10,15].

Despite these advances, progress has been uneven. While numerous initiatives have demonstrated local success, sustained system-level transformation of the CLE has proven difficult, with many interventions remaining isolated, context-specific, or insufficiently aligned with broader educational and healthcare priorities [14]. A stronger, more effective CLE is needed now more than ever in an era when learners often receive insufficient coaching and feedback, learners may perceive assessment as punitive rather than developmental, and faculty may fear reprisal or emotional harm from honest feedback [1,7,13]. Timely, actionable feedback existing within an intentionally designed program of assessment is known to be critical to establishing a strong CBHPE program [3]. However, the factors outlined above can and often do lead to a climate of mutual apprehension [12,20].

In sum, as the delivery engine for the core components of CBHPE, the CLE in its current form, still requires significant work and must evolve to fully realize the potential of CBHPE. Despite growing interest in CLEs, no contemporary framework exists to align CLE reform with the goals of CBHPE. To address this gap, we convened an international group of experts to identify priorities and generate a collective vision for CLE reform.

## Approach

This paper represents a consensus-informed call to action developed during a two-day international summit of the International Competency-Based Health Professions Education Collaborators, an interdisciplinary group of educators, researchers, institutional leaders, and CBHPE scholars representing medicine, nursing, dentistry, pharmacy, veterinary medicine, and other health professions. The summit was held at Stanford University (Stanford, California, USA) in February 2025.

Prior to the summit, authors AA, KL, and EA prepared a written primer synthesizing relevant literature, conceptual frameworks, and implementation challenges related to CBHPE and the CLE (Appendix 1). The primer was distributed to establish a shared conceptual foundation and common language for discussion. Participants voluntarily enrolled in the CLE-focused working group, one of several thematic sessions offered during the summit.

Two facilitated sessions, each approximately three hours in duration, were held on consecutive days. Participants first generated ideas individually before engaging in small-group and plenary discussions to elaborate, clarify, challenge, and refine emerging concepts. Session notes were recorded by facilitators and subsequently consolidated into summary documents.

Following the summit, the author group met virtually over a three-month period to review and refine the outputs from the working sessions. Ideas generated during the sessions were organized into provisional thematic groupings and iteratively compared, merged, and refined. Candidate themes were discussed in relation to their relevance across professions and training contexts, their importance for CBHPE implementation, and their potential impact on learner development and patient care. Through repeated discussion, prioritization, and consensus-building, five interdependent domains were identified as foundational priorities for advancing CLE reform in support of CBHPE. Detailed session summaries illustrating this developmental process are provided in Appendix 2.

## Resultant Priorities

We identified 5 priorities for transforming the CLE to support CBHPE (Table 1): 1) promoting and supporting psychological safety within the CLE, 2) prioritizing the teaching environment through enhancing faculty well-being, recognition, and creating sustainable teaching capacity, 3) capitalizing on desirable difficulty and productive struggle when the learning and teaching environments are both safe, 4) recognizing interdependence and collectivism as required cultural paradigm shifts, and 5) aligning healthcare and education system goals and priorities.

**Table 1:** Summary of key priority action items.


PRIORITY	KEY ACTIONS

#1: Psychological Safety & Growth Mindset	Integrate vulnerability and adaptability coaching into faculty and learner development (storytelling, uncertainty-simulation). Provide protected time/incentives for peer observation focused on safety behaviors. Embed learner-reported psychological safety metrics into program dashboards, linking results to recognition and support. Explicitly teach and model growth mindset principles, pairing them with feedback literacy.

#2: Teaching Environment & Trust	Recognize, support and value clinical teachers as essential system assets. Address workload strain and protect time for teaching. Mitigate misuse of evaluation systems and promote trust through clear feedback norms. Apply Quality Improvement (QI) approaches that integrate patient care and teaching priorities.

#3: Desirable Difficulty & Challenge	Educate learners on the science of productive struggle and desirable difficulty and set expectations for training. Dispel myths that psychological safety equals comfort or conflict avoidance. Incorporate evidence-based strategies (retrieval practice, spaced repetition, interleaving) into clinical teaching. Train supervisors to coach through challenge rather than avoid it, maintaining accountability without punitive overtones.

#4: Interdependence & Collectivism	Reward followership, shared leadership, and collaborative achievements alongside individual excellence. Embed team-based assessment and feedback into training, emphasizing reciprocal growth. Foster interdisciplinary debriefing and after-action reviews modeled on high-reliability teams. Use vulnerability as a lever for building trust and collective accountability.

#5: Stakeholder Alignment & System Integration	Build formal partnerships between education leaders and health system leadership to align goals. Measure and monetize the value of strong CLEs (e.g., recruitment/retention, patient outcomes). Engage learners directly in health systems improvement projects. Apply familiar organizational improvement frameworks (e.g., Learning Health System, Deliberately Developmental Organization) to embed continuous learning.


### Priority 1: Promoting and supporting psychological safety within the CLE

Defining, promoting, and supporting psychological safety are key in developing a CLE capable of delivering the CBHPE core competencies. The concept of “psychological safety” was mainly developed by Edmondson, who defined it as “a shared belief held by members of a team that the team is safe for interpersonal risk taking” [21]. Vulnerability can thus only thrive where psychological safety exists.

This is important because the CBHPE core components such as tailored learning and authentic feedback require a significant degree of vulnerability. Such reform extends beyond curricular adjustment and demands a cultural transformation in which supervisors model the behaviors, mindsets, and interpersonal norms that learners then internalize [22]. Central to this transformation is the deliberate demonstration of vulnerability by supervisors, which often runs counter to the current norms of perceived infallibility. When supervisors and other members of healthcare teams openly acknowledge uncertainty, narrate their reasoning, invite critique of their clinical plans, and share stories of past missteps and the insights gained, they normalize this fallibility as an inevitable companion to growth [23].

Simultaneously, this modeling by supervisors demonstrates how to cope with unpredictable clinical pressures, external systemic pressures, evolving evidence, and changing patient needs. It signals to learners that maturity in healthcare is less about omniscience and more about iterative sense-making and team-based problem solving, which leads to the transformation of the CLE into a competency-based learning community [3,24]. Without this mindset, admissions of doubt are read as weakness rather than courageous curiosity, which in turn likely stunts competency-based growth.

Having a shared mental model and language around psychological safety is a central pillar of CLE reform and is inseparable from faculty and learner development in a competency-based system. Faculty and learner development must move beyond sporadic workshops to longitudinal communities of practice that pair learners with junior and senior educators in mutual mentorship while establishing structured practices that support psychologically safe interactions. These practices include feedback conversations that clearly distinguish behavior from identity, rituals that acknowledge and celebrate near-miss reporting, and inclusive strategies that elevate marginalized voices in clinical discussions [23,25].

Such practices are foundational to equity in CBHPE, ensuring that all learners are met where they are and have access to feedback and opportunities for growth that reflect their individual developmental trajectories. Institutional leadership plays a critical role in this transformation by actively recognizing and rewarding supervisory behaviors that promote inclusion and psychological safety, thus reinforcing the values of equitable, competency-based education within the CLE. Over time, these micro-moments can coalesce into a macro-culture where curiosity outcompetes perfectionism and learners no longer feel that they must “perform” for supervisors and assessors [7,26]. These collective experiences can serve to build trust amongst learners, which is a critical element for the successful implementation of CBHPE – one that is often missing in the CLE, particularly among learners from groups underrepresented in healthcare [27].

Finally, a psychologically safe CLE can foster an intentionally nurtured growth mindset among learners and supervisors. The belief that abilities can be developed through effort, learning, and persistence aligns closely with CBHPE, which expects competence to develop over time through deliberate practice rather than to emerge at fixed milestones [28]. Learners with a growth mindset are more receptive to feedback, better able to integrate coaching insight [29,30,31]. Educators and organizations can cultivate this mindset by teaching the theory, facilitating meaningful feedback conversations, creating a culture of psychological safety, and encouraging self-directed learning [24,32,33,34,35]. As CBHPE continues to evolve, psychological safety and growth mindset together will remain essential enablers of high-functioning, equitable competency-based CLEs.

### Priority 2: Prioritizing the teaching environment through enhancing faculty well-being, recognition, and creating sustainable teaching capacity

Much of the prevailing educational literature, including the tenets of CBHPE, focuses organizations’ and educators’ attention firmly on the learners’ journey [3]. Here we advocate that an excellent *learning* environment requires an equally excellent *teaching* environment. Teaching in the clinical setting has arguably never felt more challenging. Changes in pedagogy, at the same time as increased demands on the healthcare system, have put more strain on clinical teachers, who in the world of CBHPE often face the burden of increased requirements to deliver workplace-based assessments, coaching, and personalized learning. All the while, learner numbers, learner expectations, and the complexity of patient care continue to increase [36]. In addition, a perceived “weaponization” of the system of feedback against clinical teachers, in response to feedback a learner may not agree with, contributes to the strain on clinical teachers, their potential for advancement, and enjoyment of their work [37,38].

Education administrators understand the difficulty in getting busy clinicians to teach, particularly in this complex environment. At the heart of clinicians’ professional identity is the duty to care for patients, and this can interfere with other essential duties such as teaching [7]. Furthermore, clinical work may drive revenue, and many healthcare systems operate a ‘fee for service’ model in which reimbursement mirrors the volume of patients for whom care is provided. Research is another traditionally competing interest, bringing prestige and academic promotion to both the individual and the institution. Clinical teachers must answer to multiple individuals and competing interests [39].

CBHPE’s learner-centered approach must be reconciled with the realities of trust, risk, and responsibility in clinical care. Tensions between care for patients and exposing them to an inexperienced or even struggling learner are relatable to all clinicians. There are reciprocal relationships, and each player has their own set of personal vulnerabilities. Tensions such as these can lead to learner neglect or sidelining [40].

For institutions, faculty recruitment, development, and retention can be challenging, with workforce shortages exacerbated by an increasing number of clinicians choosing to work part-time [41]. Addressing these challenges requires more than recruiting additional teachers; it requires understanding the realities of the clinical teaching workforce and intentionally designing systems that resource, support, and reward teaching as a core institutional mission.

Supporting the clinical teaching workforce requires more than asking clinicians to do more with less. Institutions must deliberately create conditions that make teaching feasible, valued, and sustainable. This includes providing protected time for teaching activities, reducing administrative burden associated with assessment and documentation, investing in longitudinal faculty development programming, and creating systems that recognize educational contributions in promotion, compensation, and leadership pathways. Educational excellence should be rewarded with the same intentionality traditionally afforded to clinical productivity and research achievement. Equally important is ensuring alignment between educational and healthcare system leaders such that teaching is viewed not as competing with patient care, but as an essential component of delivering high-quality healthcare and preparing the future workforce. By creating structures that support, recognize, and develop clinical teachers, institutions can strengthen both faculty well-being and the long-term sustainability of CBHPE.

### Priority 3: Capitalizing on desirable difficulty and productive struggle when the learning and teaching environments are both safe

Vygotsky’s Zone of Proximal Development offers a foundational lens for understanding the dynamic interplay between support and challenge in learning [42]. This calibrated zone of productive struggle is where deep, transformative learning occurs. Closely aligned with this is the concept of *desirable difficulty*, which refers to learning tasks that require considerable cognitive effort and, when appropriately designed, enhance long-term retention and performance [43]. Critically, the educator’s role is not to eliminate difficulty in the pursuit of comfort or likeability but to intentionally design learning experiences that target the optimal challenge point, meaning tasks that are difficult enough to promote growth but not so overwhelming as to induce unproductive frustration, disengagement or lack of safety [44,45]. Yet, this “sweet spot” varies across learners and contexts, requiring individualization and adaptive teaching.

Both productive struggle and desirable difficulty are essential for the development of adaptive expertise, defined as the ability to apply knowledge flexibly to novel situations [24,28,43,44]. CBHPE, with its emphasis on authentic performance and individualized progression, depends upon learners encountering and working through these developmental growth edges [46,47]. However, challenge without adequate support may be experienced as threat, while support without challenge can result in stagnation. Psychological safety and desirable difficulty are therefore not opposing forces but complementary conditions that enable meaningful learning and growth.

The concepts presented in this section are perhaps best synthesized in the essence of the Adaptive Teacher [48] and the Master Adaptive Clinician Educator [49], who flexibly adjust their approach based on learner needs and context while aligning their teaching, feedback, and learning environment with evidence-informed principles. Supervisors must be adaptive not only with what they teach but in how and when they offer support versus challenge. Figure 1 provides examples of activities that either promote or hinder learning based on Daloz’s framework [50]. While teachers cannot force learner change, they can deliberately adjust mindset, language and teaching behavior to create psychologically safe learning environments (but not without discomfort) that are simultaneously accountable (without being punitive) and challenging (without being overwhelming). The Master Adaptive Clinician Educator framework thus offers a promising guide for operationalizing desirable difficulty in clinical education, supporting both individual learner growth and broader educational transformation to achieve the goals of CBHPE [49].

**Figure 1:**
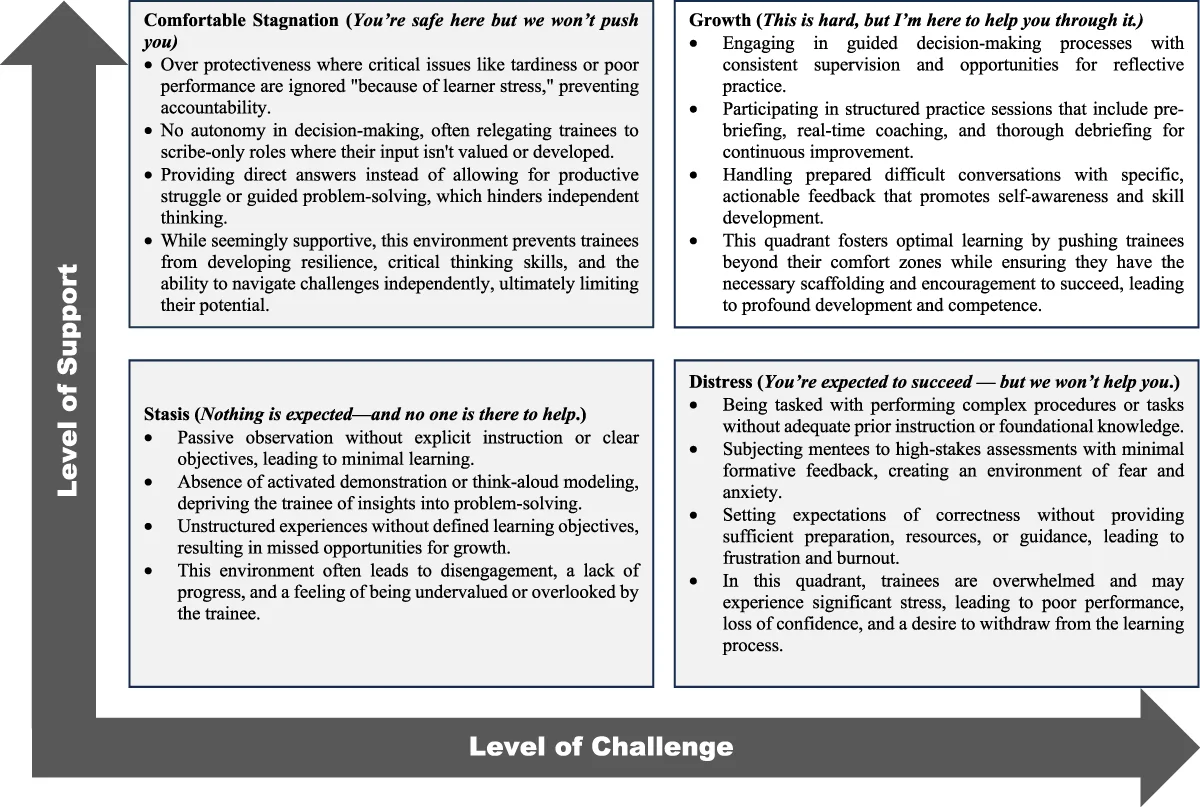
Application of Daloz’s developmental model [50] to the teacher–learner relationship illustrating supervisory activities related to competency development.

### Priority 4: Promoting interdependence and collectivism as required cultural paradigm shifts

The CLE is often discussed in terms of culture, structure, and relationships, but what underpins these dimensions is the prevailing paradigm that shapes how learning is viewed [5]. Historically, HPE has been shaped by individualism and independence: we train, assess, license, and promote individuals [51]. However, healthcare is delivered by teams, and modern systems demand interdependence, shared accountability, and collective competence [52,53]. Indeed, a group of competent individuals does not always demonstrate collective competence as a team. Thus, a misalignment exists between how care is delivered (teams) and how education is conceptualized (individual competence). Within CBHPE, this tension becomes particularly evident, as learners are expected to develop competencies in authentic team-based settings while being evaluated through methods that often emphasize individual performance, potentially undermining the collaborative mindset essential for clinical practice [7,54,55].

Collectivism and interdependence offer alternative lenses through which we can view the CLE [56,57,58]. These constructs emphasize connectedness, shared goals, and reciprocal growth [59,60]. Within collectivist CLEs, teaching is reframed not for individual performance but as relational practice; belonging is not a byproduct but a prerequisite for learning [27,61]. This shift is not merely philosophical, but rather, it is necessary for the high-functioning teams we aspire to train within [62]. High-performing teams are not only collaborative but deeply committed to each other’s growth [63]. A CLE infused with interdependence thus become fertile ground for psychological safety, feedback literacy, and professional identity formation to flourish [5,13,18]. Each of these is critical to CBHPE implementation and sustainability.

Interdependence also reconfigures how we view vulnerability: not as weakness but as the engine of trust, which is critical to the authentic coaching, feedback, and assessment so integral to CBHPE [7,64]. Importantly, this reframing does not suggest that independence should be eliminated from training. Instead, it invites a cultural paradigm shift in which independence is reconsidered not as the ultimate end goal, but as a necessary developmental step within a broader trajectory toward interdependence. As mentioned in the psychological safety section above, when educators model candor and admit uncertainty, they create space for learners to do the same. This is how CLEs can align with CBHPE principles, where zero-stakes coaching gains traction and assessment becomes a process of learning rather than judgment, a central tenet of CBHPE [65]. Interdependent CLEs recognize that feedback is not a transaction between expert and a novice but a shared endeavor among partners in care and learning [63,66]. Finally, interdependence acknowledges the interplay among team members and seeks to assess the dynamic, group-based nature of healthcare delivery and education accurately and authentically [67].

To operationalize this shift to collectivism and interdependence and thus create the cultural conditions necessary for CBHPE implementation and sustainability, supervisors should be trained to model collaboration, vulnerability, and feedback-seeking behaviors. Followership, defined as the active and intentional contribution to shared goals through collaboration, accountability, constructive challenge, and appropriate deference within a leadership process, should be explicitly taught alongside leadership [68]. Followership, shared leadership, and collaborative effort should be explicitly recognized and rewarded, not merely rhetorically. In turn, assessment systems, while continuing to promote and ensure individual-level growth through personalized feedback, must also evolve to capture individual contributions to teams and team-based interdependence [54]. Much like high-performing teams in other fields where radical candor and open critique are expected (regardless of rank) to strengthen mutual trust and collective accountability, healthcare teams should too foster a culture in which honest feedback is offered and received as an act of mutual care, reinforcing interdependence, shared responsibility, and the safety that allows individuals to be vulnerable for the benefit of the team [63,64,69].

### Priority 5: Aligning healthcare and education system goals and priorities

Historically, structured education has been carved out of the clinical environment with educators often not involved in the financial, operational, or political aspects of the clinical delivery system. However, a core principle of CBHPE is that the needs of patients and communities define desired educational outcomes [47]. Meeting this goal demands that educators become more directly involved in the environmental conditions of the systems in which they find themselves.

To align educational and health systems goals, it is important to demonstrate to stakeholders how the educational mission, including its supervisors and learner workforce, does not detract from, but is indeed critical to, achieving health system goals. Quality, safety, patient experience, and clinician well-being all benefit from educational interventions. Educators must work to formally measure and demonstrate the financial impact of a productive and reinforcing CLE (e.g., improved recruitment and retention of supervisors and team members, improved patient outcomes, greater efficiency, and reduced costs), thus improving the *return on investment* in CBHPE.

Education programs should more explicitly utilize the healthcare system itself as a formal classroom to illustrate core concepts of health systems science, focusing on *how* the health system fulfills its mission to care for patients and communities [70]. Together, health systems and education programs should consider how to formally engage learners in quality and patient safety initiatives, rather than assuming trainees will passively absorb these competencies [71,72]. There are increasing examples of partnerships in which learners, often in interprofessional teams, lead projects to address needs identified by health systems and/or individual providers [73,74]. Such “value-added” roles can have real impact on system performance, reinforcing the idea that the presence of learners makes a system stronger [75]. To support CBHPE, educators should collaborate with affiliated health system leaders to align incentives locally, actively engage learners in health systems improvement initiatives, and ensure that learners are formally recognized for their contributions.

Existing frameworks of organizational improvement can facilitate collaboration between educators and health system leaders to create CLEs that support continuous competency development for all team members, regardless of profession or level of training. The U.S. National Academy of Medicine Learning Health System framework articulates core commitments that systems make to patients, promising to provide care that is engaged, safe, effective, equitable, efficient, accessible, accountable, transparent, secure and adaptive [14,76]. These same principles should apply to the learning experience of every professional within the system.

Another useful model is the Deliberately Developmental Organization (DDO) [77]. Alignment of learning and health system goals relies upon each team member’s ability to surface uncertainties, exposing personal areas for growth while promoting quality and safety – truly the definition of a healthy CLE in which everyone thrives. The DDO formalizes and normalizes the need for continuous learning and the possibility of a safe CLE, which is critical to supporting CBHPE at all levels [12]. As such, leaders of education programs must build relationships with the teams in affiliated health systems charged with continuous improvement. By aligning with models familiar to health system leaders, educators can make the case that all workers in a health system are learners. Such conversations will be even more impactful if education leaders collaborate across health professions, acknowledging a shared stake in the clinical work and learning environment to promote CBHPE and, eventually, excellence in all.

## Conclusion

The future of CBHPE depends on the environments in which it is delivered. Without deliberate and timely reform, the promise and potential of CBHPE will remain unrealized. CBHPE can only thrive when the CLE becomes a space defined by trust, accountability, and collective growth. True reform requires a cultural shift that embraces vulnerability, coaching and feedback, and collaboration as essential to professional development. Supervisors must be supported in balancing safety with challenge, and educational and healthcare leaders must align their goals so that learning environments directly enhance patient care, staff well-being, and system improvement.

Importantly, these five priorities should not be viewed as independent initiatives, but as interdependent elements of a broader system. Psychological safety, teaching capacity, desirable difficulty and productive struggle, interdependence, and stakeholder alignment reinforce one another and collectively shape the effectiveness of the CLE. Together, they complement existing efforts to improve CLEs while providing a contemporary framework for advancing CBHPE. By intentionally cultivating these conditions, we can transform the CLE from a passive backdrop into an active catalyst for learning. The responsibility is shared; the time is now.

## Additional File

The additional file for this article can be found as follows:

10.5334/pme.2548.s1 Appendices. Appendix 1 and 2.

## Artificial Intelligence

ChatGPT 5.5 (OpenAI) was used for reference formatting and bibliography consistency checks. All outputs were reviewed, verified, and edited by the authors. The authors take full responsibility for the accuracy and integrity of all content.
