# Supplementary material for: No Environment, No Education: Honoring the Clinical Learning Environment as the Engine of Competency-Based Education – A Call to Action
Source: Perspect Med Educ. 2026 Sep 9;15(1):771–82. doi: 10.5334/pme.2548 (PMC13629532; doi:10.5334/pme.2548)
Supplement: Supplementary file 1 [file pme-15-1-2548-s1.pdf]

Appendix 1.

Part 1: What Do We Know So Far?

Clinical Learning Environments Within the Context of Competency-Based Education

Introduction to Competency-Based Education (CBE)

Competency-Based Education (CBE) represents a transformative approach in medical education, emphasizing predefined competencies that align with the desired outcomes of medical training. Unlike traditional education, which often progresses based on time-bound curricula, CBE focuses on demonstrating mastery of essential skills, knowledge, and behaviors critical to effective medical practice (Ferguson et al., 2017; Ryan et al., 2023).

CBE employs a criterion-referenced assessment strategy, where learners are evaluated against fixed benchmarks rather than against peers, ensuring consistent and equitable progression based on competency attainment (Ryan et al., 2023; Torre et al., 2021).

The Clinical Learning Environment (CLE): Definition and Importance

The CLE encompasses the social, structural, and cultural contexts where medical education occurs. It includes physical and digital spaces, interpersonal interactions, and organizational practices that shape the experiences of learners and educators. A well-structured CLE supports learning, fosters professional development, and enhances both patient safety and healthcare outcomes (Hall et al., 2023).

The CLE directly influences the achievement of competencies by providing learners with real-world contexts to apply knowledge, hone skills, and adopt professional behaviors. Effective CLEs:

- Promote inclusivity and psychological safety to optimize learning experiences (Hall et al., 2023).
- Enable longitudinal assessments through diverse evaluation methods, such as portfolios and workplace-based observations (Torre et al., 2021; Heeneman et al., 2021).
- Encourage leadership development and cultural humility among educators and learners (Ferguson et al., 2017; Hall et al., 2023).
- Address power dynamics: hierarchical and systemic inequities that may hinder learning and collaboration (Hall et al., 2023; Torre et al., 2021).
- Promote strong feedback culture: Incorporating continuous, meaningful feedback as a core component to promote growth (Torre et al., 2021; Heeneman et al., 2021).

Assessing the Educational Environment

The assessment of educational environments in medical education is critical for understanding and enhancing the quality of training and its influence on student outcomes. Measuring the educational environment allows institutions to gauge factors like student satisfaction, engagement, and success. Key instruments for these assessments include tools such as the Dundee Ready Education Environment Measure (DREEM), Postgraduate Hospital Educational Environment Measure (PHEEM), and Clinical Learning Environment and Supervision (CLES) scales. These tools evaluate domains like personal

development, goal direction, relationships, and systemic structure, which significantly impact the learner's experience (Soemantri et al., 2010; Schönrock-Adema et al., 2012).

Effective assessment relies on valid and reliable instruments tailored to specific educational settings. For example, DREEM is widely used for undergraduate settings, while PHEEM is better suited for postgraduate environments. These instruments also highlight the importance of psychological safety, role clarity, feedback mechanisms, and social support. Applying theoretical frameworks like Moos' three domains—personal development, relationships, and system maintenance—has further refined the focus of educational environment measures, ensuring comprehensiveness and applicability across contexts (Schönrock-Adema et al., 2012).

Challenges in Integrating CLE into CBE

The transition to CBE has encountered resistance rooted in traditional norms, such as time-bound progression and subjective assessments (Ferguson et al., 2017; Ryan et al., 2023). Key barriers include:

1. **Cultural inertia**: Long-held beliefs about education that prioritize summative evaluations over developmental assessments (Ferguson et al., 2017).
2. **Systemic hierarchy**: Entrenched power asymmetries that discourage open communication and hinder collaboration (Hall et al., 2023).
3. **Assessment methods**: Persistent reliance on norm-referenced grading, particularly for residency selection, which conflicts with CBE principles (Ryan et al., 2023; Torre et al., 2021).

4. **Clinical service over learning**: often, the learning environment is negatively impacted by service pressures and delivery of care is prioritized over learning/teaching, learner psychological safety, etc.

Part 2: What Strategies Have We Tried So Far? Framework-based Strategies to Improve Clinical Learning Environments

We now know that improving CLEs requires a multifaceted approach that integrates cultural, organizational, and systemic changes. By fostering inclusivity, embedding health-promoting principles, leveraging data, and promoting interprofessional collaboration, institutions can create environments that support the growth of learners and enhance patient care. Drawing insights from previous research, the following strategies have been identified to optimize CLEs:

1. **Curriculum Integration:** Designing curricula that align with CLE objectives and leverage real-world clinical settings for competency assessment (Lockyer et al., 2017; Ryan et al., 2023).

2. **Foster Psychological Safety**

- Promote environments where learners and faculty feel safe to express uncertainties and ask questions without fear of judgment or ridicule (Hall et al., 2023).
- Develop policies and practices that encourage open communication, inclusivity, and the reduction of power hierarchies within medical teams (Fostering a Health-Promoting Learning Environment, 2023; NCICLE, 2021).

3. **Feedback Literacy**: Teaching learners and educators to provide and utilize feedback effectively to inform learning and practice (Torre et al., 2021; Heeneman et al., 2021).

4. Embed Health-Promoting Principles

- Adopt frameworks like the Okanagan Charter, which integrates health promotion into all aspects of the academic and clinical environment (Do et al., 2023).
- Establish strategies such as embedding health in institutional policies, developing supportive spaces, and prioritizing well-being for students and faculty (Do et al., 2023).

5. Leverage Learning Health System (LHS) Frameworks

- Implement core LHS principles such as continuous learning, innovation, and equity in clinical operations (Rosenthal et al., 2023).
- Use robust data systems to support knowledge generation, patient care improvement, and learner engagement in evidence-based practices (NAM, 2024).

6. Promote Interprofessional Collaboration

- Design CLEs that emphasize interprofessional teamwork, incorporating learners from various health professions into collaborative care models (NCICLE, 2021).
- Strengthen leadership and supervision to create cohesive, high-functioning teams that balance learning with patient care delivery (NCICLE, 2021).

7. Address Equity, Diversity, and Inclusion (EDI)

- Prioritize EDI initiatives by revising curricula to eliminate systemic biases and integrating these considerations into accreditation processes (Hall et al., 2023; Fostering a Health-Promoting Learning Environment, 2023; Torre et al., 2021).
- Encourage representation and cultural humility to ensure all learners and patients feel valued and respected (Hall et al., 2023).

8. Utilize Data and Digital Tools

- Enhance CLE effectiveness by digitizing and integrating assessment data into organizational decision-making processes (Thoma et al., 2023).
- Use data visualization and analytics to identify areas for improvement, track learner progress, and inform feedback mechanisms (Thoma et al., 2023).

9. Implement Programmatic Assessment

- Programmatic assessment is a cornerstone of CBE, integrating multiple data points across time to evaluate learner progression comprehensively. It emphasizes formative feedback, data aggregation, and decision-making based on triangulated evidence (Torre et al., 2021; Heeneman et al., 2021). This approach aligns with CLE principles by fostering continuous improvement and supporting personalized learning plans.
- Develop longitudinal assessment strategies that emphasize formative feedback, triangulation of data, and comprehensive evaluation of learner progress (Torre et al., 2021).
- Align assessment practices with CLE objectives to foster a culture of continuous improvement and accountability (Hall et al., 2023).

10. Engage Leadership and Stakeholders

- Secure organizational commitment and leadership buy-in to implement CLE reforms effectively (NAM, 2024).
- Establish collaborative structures and strategic communication pathways to ensure stakeholder alignment and sustained efforts toward CLE optimization (Do et al., 2023; NCICLE, 2021).
- Building leadership capacity and providing training on CBE principles are critical for fostering innovation in CLE (Ferguson et al., 2017; Torre et al., 2021).

Part 3: But How Can We Be Even Better?

Despite everything we know above, the **Clinical Learning Environment (CLE)** is slow to evolve. Various pockets of success have been described here and there in the literature, yet system-wide change remains elusive.

CBME "implementation" has become about "compliance" - and this is the source of cynicism and "low fidelity/integrity". The pockets of success where things have gone well (E.g. Sue HM Rheum EPAs/CC, CBVE) do not come from a place of "forced compliance" - it came from shifting the focus away from the minimum floor, and towards the height of the ceiling, "excellence," if you will - Growth Mindset can only happen if the target is just BEYOND reach, not immediately attainable.

The CLE serves as the **delivery vehicle** for the philosophies of **Competency-Based Education (CBE)**. The effectiveness of this delivery is determines whether CBE arrives **under fire in a Humvee** (chaotic delivery: all the eggs in the carton cracked, not-as-intended low fidelity/integrity implementation) or in a **controlled, comfortable SUV** (intentional delivery: eggs intact, high integrity/fidelity implementation).

Faculty Must Drive: Growth Mindset First

Faculty members are the **drivers** of the CBE vehicle. Subsequently, their modeling of **growth mindset** is essential. Key aspects include:

- **Modeling vulnerability** and avoiding performative teaching – Faculty should **share personal experiences, display candor, and model learning behaviors** rather than simply putting on a show. When faculty focus on performance over authenticity, learners may mirror this behavior, leading to assessment (e.g. EPA (Entrustable Professional Activities) **weaponization** and a **performance-driven** rather than a **mastery-driven** mindset.
- As Faculty, we must make a decision about how we are going to conceptualize the dirty word: "Growth Mindset": **Is competency relative, or is it absolute?** We cannot continue with norm-referenced grading (absolute), yet extoll Vygotskyan proximal-zone-of-development-type growth mindset (relative) as the pathway to competency. The tension between the two is where learner cynicism is born – "you're telling me I should ask silly questions, but when I do, I get a silly grade."
- **The Unfortunate Acculturation to Performance Mindset:** Even high-level athletes who enter medicine shift toward a performance-driven mindset to survive and advance. Perhaps the role of the **Master Adaptive Teacher** is just as crucial as that of the Master Adaptive Learner.

The Renaissance of Challenge in the Learning Environment

- **Unsafe and Uncomfortable are not the same. Discomfort is necessary for growth** but must exist within a **psychologically safe environment** (eustress vs distress).
- **Safety as a compound-interest phenomenon:**
    - First-year learners need safety to **voice uncertainties**

    - Final-year residents feel safe to **ask the hard questions** that directly impact their clinical competency and readiness for independent practice.
    - **How can we get junior learners there faster?**
- **Assessment:** discrepancies between learner vs staff-triggered assessments
    - **Learner-driven assessments** – Most learners are entrusted but receive minimal structured feedback.
    - **Staff-driven assessments** – Faculty provide **large amounts of feedback**, yet many are **only partially entrusted** in certain competencies.
    - **Zero-stakes coaching** (not just "formative" or "low-stakes") may be the strongest model for true growth.
- **Trust is key:** "Eyes on me, don't mind the noise behind us." Even within **unsafe organizations**, **safe micro-environments** can be cultivated—these may be the foundation of broader **organizational safety.**

What have been some barriers to systematic CLE improvement?

- Most available literature is **narrative-based** rather than **evidence-based**—valuable, but difficult to implement systematically.
- Lack of **incentives** – There is no clear **reward or punishment** for CLE change.
- Could we **"dollarize" the value of a strong learning environment** (or the cost of a poor one) to drive institutional change and stakeholder commitment? Other areas have already done this well: burnout is costly, "compassionomics."
- The small pockets of success thus far have been either 1) derived from business/management theory, 2) demonstrated monetary value, or 3) were patient-centric outcome-based interventions where the CLE changed as a byproduct.

More Frameworks and Models to Consider

1. **Learning Organization (Peter Senge)**

- Origin: Peter Senge, *The Fifth Discipline* (1990)
    - Organizations should continuously learn and adapt through: Systems thinking, Personal mastery, Shared vision, Team learning, Mental models

2. **Followership / Servant Leadership**

- Leaders should serve their teams, prioritizing their growth and well-being.
- A developmental approach to leadership fosters long-term engagement and competency.

3. **The iPACE Model**

- The iPACE Model (Interprofessional Patient-Aligned Care Team) is a structured approach to interprofessional bedside rounding that enhances team collaboration, patient-centered care, and communication in hospital settings. It aims to improve patient outcomes by integrating various healthcare professionals into the rounding process.

4. **Communities of Practice (CoP)**

- Communities of Practice (CoP) Forums enhance the Clinical Learning Environment (CLE) by fostering a collaborative, supportive, and reflective culture among healthcare professionals and learners. They create a structured space for knowledge-sharing, professional development, and interdisciplinary teamwork, which positively influences clinical training and patient care.

5. **Interprofessional Clinical Learning Environment (IP-CLE)**

- IP-CLE ensures high-quality, competency-driven healthcare education. It fosters critical thinking, decision-making, and interprofessional collaboration, ultimately improving patient care and professional development.
- 5-step implementation framework:
  - Gap Assessment: Identifying learning needs and competency gaps.
  - Stakeholder Involvement: Engaging educators and professionals in planning.
  - Tailored Learning: Developing targeted education strategies.
  - Competency Validation: Assessing skill application at the point of care.
  - Debriefing and Evaluation: Reviewing outcomes for continuous improvement.

6. Deliberately Developmental Organizations (DDO)

- Rooted in organizational development, the DDO framework emphasizes three key dimensions: developmental communities, aspirations, and practices within graduate medical education.
- These dimensions encourage collective responsibility, where faculty and trainees share goals and view weaknesses as growth opportunities (Miller et al., 2023).
- By addressing hierarchical and relational challenges, DDO principles support both individual and organizational resilience in complex healthcare systems (Miller et al., 2023).

7. Technical Guides on CLEs

- Saint Louis: https://www.slu.edu/medicine/about/faculty-resources/cedar/posts/clinical-learning-environment.php
- Australia: https://www.health.qld.gov.au/__data/assets/pdf_file/0029/693335/cle-review.pdf
- Saskatchewan: https://medicine.usask.ca/documents/pgme/learning-environment.pdf

References

- Ferguson, P. C., Caverzagie, K. J., Nousiainen, M. T., Snell, L., & ICBME Collaborators. (2017). Changing the culture of medical training: An important step toward the implementation of competency-based medical education. *Medical Teacher*, *39*(6), 599-602. https://doi.org/10.1080/0142159X.2017.1315079
- Lockyer, J., Bursey, F., Richardson, D., Frank, J. R., Snell, L., Campbell, C., & ICBME Collaborators. (2017). Competency-based medical education and continuing professional development: A conceptualization for change. *Medical Teacher*, *39*(6), 617-622. https://doi.org/10.1080/0142159X.2017.1315064
- Hall, J., Atkinson, A., Chan, M.-K., Tourian, L., Thoma, B., & Pattani, R. (2023). The Clinical Learning Environment in CanMEDS 2025. *Canadian Medical Education Journal*, *14*(1). https://doi.org/10.36834/cmej.75537
- Ryan, M. S., Lomis, K. D., Deiorio, N. M., Cutrer, W. B., Pusic, M. V., & Caretta-Weyer, H. A. (2023). Competency-based medical education in a norm-referenced world: A root cause analysis of challenges to the competency-based paradigm in medical school. *Academic Medicine*, *98*(11), 1251-1260. https://doi.org/10.1097/ACM.0000000000005220
- Torre, D., Rice, N. E., Ryan, A., Bok, H., Dawson, L. J., Bierer, B., Wilkinson, T. J., Taith, G. R., Laughlin, T., Veerapen, K., Heeneman, S., Freeman, A., & van der Vleuten, C. (2021). Ottawa 2020 consensus statements for programmatic assessment: Implementation and practice. *Medical Teacher*, *43*(6), 651-660. https://doi.org/10.1080/0142159X.2021.1956681
- Heeneman, S., Bierer, B., Wilkinson, T. J., Bok, H., & van der Vleuten, C. (2021). Performance assessment in competency-based medical education: Insights from the Ottawa consensus. *Medical Teacher*, *43*(6), 671-677. https://doi.org/10.1080/0142159X.2021.1956681

- Miller, K. A., Nagler, J., Wolff, M., Schumacher, D. J., & Pusic, M. V. (2023). It takes a village: Optimal graduate medical education requires a deliberately developmental organization. *Perspectives on Medical Education*, *12*(1), 282–293. https://doi.org/10.5334/pme.936
- Schönrock-Adema, J., Bouwkamp-Timmer, T., van Hell, E. A., & Cohen-Schotanus, J. (2012). Key elements in assessing the educational environment: Where is the theory? Advances in Health Sciences Education, 17(5), 727–742. https://doi.org/10.1007/s10459-011-9346-8
- Soemantri, D., Herrera, C., & Riquelme, A. (2010). Measuring the educational environment in health professions studies: A systematic review. Medical Teacher, 32(12), 947–952. https://doi.org/10.3109/01421591003686229
- Do, V., Lewis, M., Goldstein, C., & Sonnenberg, L. K. (2023). Fostering a health-promoting learning environment in medical education: Adapting the Okanagan Charter for administrators and medical educators. *Academic Medicine.* https://doi.org/10.1097/ACM.0000000000005159
- Hall, J., Atkinson, A., Chan, M.-K., Tourian, L., Thoma, B., & Pattani, R. (2023). The clinical learning environment in CanMEDS 2025. *Canadian Medical Education Journal, 14*(1). https://doi.org/10.36834/cmej.75537
- Miller, K. A., Nagler, J., Wolff, M., Schumacher, D. J., & Pusic, M. V. (2023). It takes a village: Optimal graduate medical education requires a deliberately developmental organization. *Perspectives on Medical Education*, *12*(1), 282–293. https://doi.org/10.5334/pme.936
- National Collaborative for Improving the Clinical Learning Environment (NCICLE). (2021). *Pathways to excellence: Expectations for an optimal clinical learning environment to achieve safe and high-quality patient care.* Chicago, IL: NCICLE. https://doi.org/10.33385/NCICLE.0003
- National Academy of Medicine (NAM). (2024). *Learning health system shared commitments initiative.* https://nam.edu/publications-of-the-leadership-consortium
- Rosenthal, G. E., McClain, D. A., High, K. P., Easterling, D., Sharkey, A., Wagenknecht, L. E., O'Byrne, C., Woodside, R., & Houston, T. K. (2023). The academic learning health system: A framework for integrating the multiple missions of academic medical centers. *Academic Medicine, 98*(9), 1002–1007. https://doi.org/10.1097/ACM.0000000000005259
- Thoma, B., Caretta-Weyer, H., Schumacher, D. J., Warm, E., Hamstra, S. J., & Chan, T. (2023). Becoming a deliberately developmental organization: Using competency-based assessment data for organizational development. *Medical Education Review.*

Appendix 2.

CLE DAY 1: DISCUSSION POINTS

INTRODUCTORY REMARKS

- Adversarial nature of relationship between Learner and Teacher. Learners have never been so terrified of Teachers, and Teachers have never been so terrified of Learners. The learning environment is more important than ever.
- The "Teaching Environment": we don't talk enough about what enables or promotes teaching, teaching identity, and positive interactions between Teachers and Learners. PTSD, even, on the part of teachers.
- Our current program of activities like "rounds" for example, or quick rotation of faculty, may not be promoting strong learning environments, and in fact may be harmful.
- "CBME = Programmatic Assessment", i.e. a new way of doing assessments with more administrative burden, rather than a new focus on learner's growth.
- The Educator HR production mechanism: to do this prospectively, rather than retrospectively is a new challenge.
- CBME cannot be a veneer, a cover on top of a pre-existing structure that does not support it.

AFTER ADELLE'S SECTION

- Tailored learning experiences: maybe most important, yet this is most difficult to achieve (due to many reasons, e.g. service pressures), and perhaps least implemented (e.g. in Canada). Prevented externally from structural reasons, culturally, financially. This culture trickles down to learners and their environment.
- Workers or Learners? When seen as a Worker, which they are often, it is more difficult to create Tailored Learning Experiences.
- There is growing pushback from Learners against the identity of Workers: "this is not good for my learning" and refusal to do the "Work".
- The Clinical Learning Environment often falls in between the domain of the Educators and the Administrators, and a fatalist attitude ensues: we need to be able to bridge the two worlds to affect the learning climate.
- TLE requires vulnerability and exposure with candor from the learner, as it could take years of (even direct) observation to understand and develop the optimal TLE plan. This kind of "learning plan" could be used as a guide from each Teacher.
- "Best" CLEs where learners felt most seen/known aren't necessarily the ones with the most time invested for teaching/learning. It happens in environments where learners feel deeply known and cared for, and know it is safe to ask for help, even if from an external perspective it appears "unsafe" (e.g. not home for days/weeks on end due to service demands). ?Link to "Reintroduction of Challenge" to the CLE?
- The CLE impacts everybody, not just Physicians.
- CLE tension is often a reflection of cultural tension: the Teachers and the Learners often have conflicting expectations (e.g. duty hours, manifestations of respect). Maybe the "solutions" we are prescribing are not addressing root culture, which is more difficult to change.
- Creativity: the learner with a goal in mind (e.g. getting through school, understanding what the objective is, "Doing School" book) will only do what is required and will not step outside of the track of work. Performance mindset.
- Mistakes have become individualized - normalization of mistakes, even requiring it, is critical to a safe learning environment, and removing the individual from the mistake.

- Psychological safety has become the death of feedback. Rather than confront the difficult process of growth, it has become easier to avoid for fear of treading on “psychological safety”.
- Trust: building pockets of trust little by little can lead eventually to cultural change.
- Mortality & Morbidity as an institution: original intent was for absolute exposure with absolute safety. Not always how it plays out.
- Zero tolerance is zero safety.
- How does this translate from academic centres to community-based practice, without the academic support?

AFTER KIM’S SECTION

- Culture exists because it facilitates: it is the unspoken truths and ways in which we work that meets a purpose. If it doesn’t align with CBME, we should ask ourselves: what purpose is it serving? What is it about the current culture that make it so hard to change to align with CBME?
- Systems-wise, we may have to re-consider how to prioritize and adapt Van Melle’s 5 core componetents of CBME in a realistic manner that reflects the environment it is being implemented in (e.g. learning environment, service environment).
- Not all of the core components are equal (nor perceived as equal). “Easier” components like form-based assessments often get prioritized. TLE/cultural change may be the rising tide that elevates everything else, maybe sometimes at the expense of some assessments/Outcome Competencies
- Perhaps “Alignment” is the problem. The interface between the Work and the Learning suffers from malalignment. The “Health System” needs to be engaged, as it will bring in growth/volume/allied health, and consider learners as future workforce, reflecting reality and aligning with CBME.
- Prioritization: not all components are equal. We must ask: What is the return on investment? We must align healthcare system goals with those of CBME. Productivity/patient volume for example, can be data that supports positions we must take as Educators.

DAY 1: SESSION SUMMARY

KEY THEMES OF THE DISCUSSION

1. THE LEARNING AND TEACHING ENVIRONMENT

- CLE is essential for shaping trainees' experiences, but systemic challenges often undermine its effectiveness.
- Faculty members feel unprepared or unsupported in their roles, leading to suboptimal teaching environments.
- The adversarial relationship between teachers and learners has intensified, reducing trust and discouraging open dialogue.
- Psychological safety is crucial but should not eliminate constructive feedback; rather, it should enable growth-oriented discussions.
- Faculty experience “PTSD-like” symptoms from negative teaching experiences, exacerbating burnout.

2. THE CHALLENGES OF CBME IMPLEMENTATION

- CBME is often perceived as a bureaucratic burden rather than a transformative approach to learning.
- The emphasis on documentation often overshadows meaningful educational interactions.
- Programs such as rapid faculty rotations and structured rounds may inadvertently harm the CLE by limiting relationship-building between faculty and learners.
- CBME cannot be a superficial overlay on an outdated system; it requires deep structural changes.

3. Tailored Learning Experiences (TLE) and Institutional Barriers

- TLE is a critical yet difficult-to-implement component of CBME due to service pressures and cultural resistance.
- Medical learners are often seen more as workers than students, making it harder to tailor their education.
- A growing pushback from learners against excessive service demands highlights a need for clearer role definitions.
- Institutional structures often hinder meaningful mentorship and individualized learning plans.
- The best CLEs are not necessarily those with the most time invested in teaching, but those where learners feel known, valued, and safe to ask for help.

4. Systemic and Cultural Issues in Medical Training

- CLE tension often reflects broader cultural tensions between teachers and learners regarding expectations, duty hours, and respect.
- Zero-tolerance policies can paradoxically create zero-safety environments where individuals fear admitting mistakes.
- Creativity in learning is limited by rigid performance metrics, where students focus on meeting requirements rather than engaging in deep learning.
- Mistakes should be normalized as learning tools rather than seen as individual failures.
- Psychological safety should enable constructive conflict and feedback, rather than stifling necessary conversations.

5. Bridging Education and the Healthcare System

- The CLE affects not just physicians but all healthcare workers, making interdisciplinary collaboration essential.
- The misalignment between educational goals and healthcare system priorities is a significant barrier to meaningful reform.
- The healthcare system must be engaged in discussions about aligning service demands with educational objectives.
- Competency-based errors—mistakes made even at high levels of expertise—should be studied and addressed to improve both learning and patient care.

Recommendations for Improvement

1. **Support Faculty Development**
    - Provide faculty with structured training and resources for mentorship and feedback.
    - Recognize and reward educational efforts institutionally.
2. **Foster a Culture of Psychological Safety and Trust**
    - Shift the focus from avoiding mistakes to learning from them in a collaborative manner.
    - Build trust through incremental efforts that encourage open communication.
3. **Balance Learning and Service Responsibilities**
    - Clarify the distinction between learners and workers, ensuring educational goals are not secondary to service needs.
    - Protect time for meaningful mentorship and feedback sessions.

4. **Reframe Assessment and Feedback Mechanisms**
    - Reduce the emphasis on administrative assessment and focus on formative feedback.
    - Encourage self-directed learning while maintaining institutional accountability.
5. **Integrate Educational Goals with Healthcare System Priorities**
    - Involve hospital administrators in discussions about improving the CLE.
    - Develop strategies that align workforce needs with CBME principles.

CONCLUSION

The discussion underscores the need for a cultural and systemic shift in medical education. Faculty and learners must collaborate to create an environment that fosters professional growth, supports CBME, and integrates learning with clinical responsibilities. Addressing systemic challenges through trust, support, and meaningful feedback will enhance both medical education and patient care.

CLE DAY 2: DISCUSSION POINTS

INTRODUCTORY REMARKS

- Aligning expectations between the learning environment and the teaching environment will be important.
- Caution: unproductive to catastrophize with regards to the "adversarial nature" of the learning environment. Having said that, it is true that there are increasing reports from teachers fearing reprisal or being labelled "offensive," "oppressive," and thus have stopped teaching. A productive way to describe the issue and a productive systems-wide approach that also engages leadership will be important going forward.
- There is a need to consider whom the target audience is for the scholarship that will result from this: who is this for? Maybe this is not a "solutions" piece of scholarship, but is rather a "call to action". It should include a value proposition: what would the health system get in exchange for change (return on investment)?

AFTER DIDACTIC SECTION

- Engagement of leadership to buy-in is critical to success. Not just on paper, but leadership needs to role model safety for CLE to be successful.
- Aligning health system's interests with those of a successful learning environment is important. Could we "dollarize" the value of a strong learning environment (or the cost of a poor one) to drive institutional change and stakeholder commitment? Other areas have already done this well: burnout is costly, "compassionomics."
- iPACE: not a CLE initiative, not even an education initiative. It is a patient-centric care-model shift that resulted in CLE improvement (with dollar values attached).
- Professionalism in clinical (learning) environment: patient outcomes (morbidity/mortality) are a compelling argument for professionalism.
- Unionization of trainees with an identity of "employee" changes the interpersonal dynamic and can make delivery of feedback challenging – is the feedback HR-compliant? HR becoming progressively more involved. Maybe this is a weaponization of identity: 'learner vs employee PRN' – cannot claim privilege and deny responsibility. On a larger scope, could this be a sign of fear of feedback, because feedback is perceived as unilaterally punitive?

- Conceptually, concentric circles: at its core, the dyad of Teacher/Learner, and outside of that, external factors. The model should be designed to protect the innermost core.
- What is it about the Learning Environment that we can focus on that is CBE-specific? Is there anything different about a CBE-specific CLE vision that is different than other learning paradigms? Van Melle's core components describes conceptual frameworks (e.g. mastery learning, ZPD, growth mindset etc) that realistically cannot be operationalized without a strong CLE.
- The appropriate frame/lens for this scholarship may be: "What are adaptations to the CLE that are conducive to CBE". This keeps the focus on CBE, and away from catastrophizing the negative aspects.
- Debate about whether the CLE is the 6th component, or, the foundational mechanism through which the core components are delivered, e.g.:

- The successful cases we have seen so far have a heavy slant on interprofessionality. We will need to engage allied health to create successful CLE change.
- Competition between institutions and lack of shared data for exemplars vs CLEs-in-trouble makes it further difficult to effectuate meaningful change – are we destined to repeat our mistakes over and over? NCICLE may be a way that information is shared for the common good.

DAY 2: SESSION SUMMARY

Key Themes

1. Understanding the Clinical Learning Environment (CLE) as the Core of CBME

- CLE is the foundation that delivers CBME principles, yet it remains under threat due to implementation barriers and systemic cynicism.
- Psychological safety was a recurring topic, with discussions on differentiating between feeling "safe" and feeling "comfortable."
- Faculty members experience uncertainty in their roles, leading to reluctance in teaching, while learners fear exposing their weaknesses.
- The adversarial nature of CLE, coupled with increasing faculty reluctance, has led to a decline in effective educational engagement.

2. Addressing Challenges and Gaps in CLE

- CLE often gets caught between healthcare system demands and the need for structured teaching and learning.
- Interprofessional collaboration is essential but remains underdeveloped in many settings.
- The misalignment of educational priorities with healthcare system objectives has created sustainability issues.
- There is a lack of systematic interventions that address both faculty engagement and learner development.

3. Proposed System-Based Solutions

- Leadership commitment is critical for successful CLE reforms.
- Data-driven interventions should be used to measure and refine CLE effectiveness.
- Institutions need frameworks that balance learner development with healthcare productivity.
- Defining clear expectations for faculty and learners can reduce misunderstandings and improve engagement.

Case Studies and Evidence-Based Approaches

1. **Interprofessional Bedside Rounding at Maine Medical Center:**
    - Implementation of structured bedside rounding improved patient engagement and cost efficiency.
    - Residents spent significantly more time discussing patient care in interdisciplinary settings.
    - The initiative expanded across 32 hospital units due to its success.
2. **Communities of Practice:**
    - Participation in communities of practice led to increased psychological safety and adaptive expertise among learners.
    - Faculty reported improvements in collaboration, innovation, and retention.
3. **Insights from Nursing Education:**
    - Emphasized structured communication, shared accountability, and patient-centered care.
    - Highlighted the importance of continuous learning beyond traditional stepwise approaches.

Challenges and Roadblocks

- Faculty fear repercussions from students, making them hesitant to provide direct feedback.
- Learners may resist competency-based frameworks if they perceive feedback as unsafe or punitive.
- Existing intervention models often lack rigorous evaluation and outcome measurement.
- HR and administrative policies sometimes create barriers to implementing flexible educational models.

Advancing CLE through System-Based Approaches

- Aligning medical education with healthcare delivery models is crucial for sustainability.
- AI and data analytics can facilitate real-time feedback and competency tracking.
- CLE reforms should be tied to institutional financial incentives to encourage broader adoption.
- Emphasizing the value of CLE through improved patient outcomes, reduced errors, and faculty retention can drive change.

Conclusion and Future Directions

- The discussion highlighted the need for system-wide strategies to optimize CLE for CBME.
- Institutions must integrate educational reforms with broader healthcare system priorities.
- Research should focus on validating successful CLE interventions and developing scalable models.
- Future efforts should aim at fostering a learning environment that supports both educators and learners while improving overall healthcare outcomes.

The conversation underscored that improving CLE requires a balance between education, patient care, and institutional policies, ultimately striving for a more effective and sustainable medical education system.
